# THE FL-REACH PROJECT: TRANSLATING AN EBP FOR AN OUTPATIENT CLINICAL SETTING TO REACH DIVERSE COMMUNITY MEMBERS

**DOI:** 10.1093/geroni/igz038.981

**Published:** 2019-11-08

**Authors:** Tracy C Wharton, Daniel Paulson, Rosemary Laird, Judy Clark, Gayle Shepherd, Felicia Bender

**Affiliations:** 1 University of Central Florida, Orlando, Florida, United States; 2 Advent Health Orlando, Orlando, Florida, United States

## Abstract

With more than 500,000 patients with Alzheimer’s disease, constituting nearly 10% of all cases in the US, the state of Florida spends an estimated $20 billion per year on care and treatment related to this disease. The Florida State Plan on Aging reported that 75% of informal caregivers felt that early education and training should be a high priority for the state, and that difficult behaviors and limited knowledge about dementias were among the most significant challenges that they faced. The REACH II intervention is the gold-standard for evidence-based practices that address burden, well-being, and skills training for dementia caregivers. This presentation describes the partnership of a Memory Disorder Clinic (MDC) team and two university-based researchers working to embed a modified REACH protocol into an outpatient clinic. Critical streamlined components and new material designed to innovations since the REACH trials. The FL-REACH protocol is significantly shorter, adds a structured assessment for both patient and family needs, expands to include advanced care planning and grief modules, and intentionally builds linkages to the medical care team, with a focus on outreach to diverse families. This manualized intervention is offered to families of patients diagnosed through the MDC, thus capitalizing on the rapport and trust that is built, and providing in-house opportunities to engage diverse populations with a program grounded in the evidence base. This intervention provides critical foundational training for families that will bridge to seamless team coordination in the future.

